# Supplementary material for: The CRISPR ring nuclease Csx15 oligomerises on cyclic nucleotide binding to regulate antiviral defence
Source: Biochem J. 2026 Apr 17;483(5):699–712. doi: 10.1042/BCJ20260019 (PMC13148439; doi:10.1042/BCJ20260019)
Supplement: Supplementary Figures S1-S8 and Tables S1-S2 [file BCJ-2026-0019_supp.pdf]

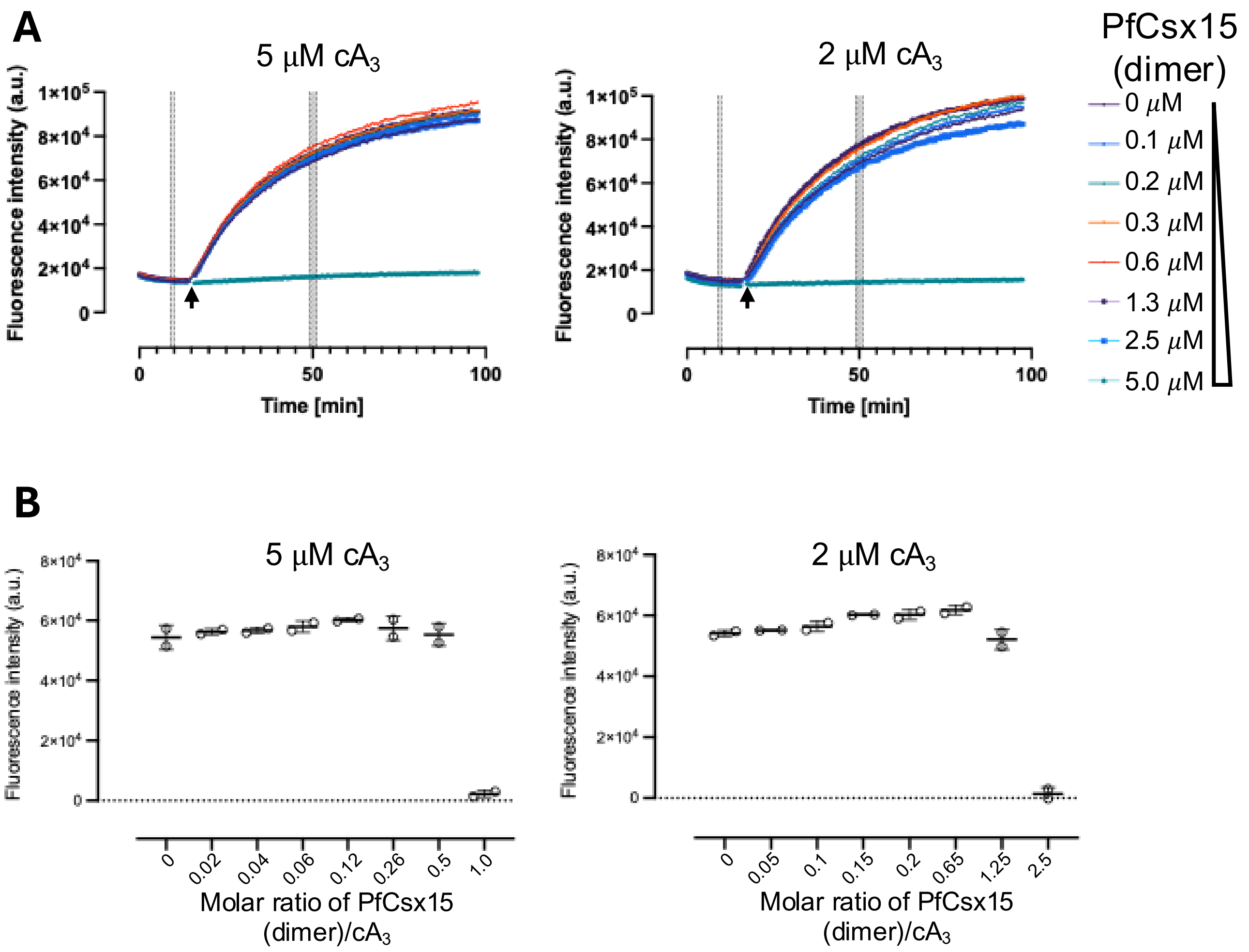

Supplementary Figure 1: PfCsx15 does not strongly inhibit the activated effector VmeNucC

A. Fluorescence signal emitted by dsDNA cleavage when DNases were presented. dsDNA substrates (100 nM) were incubated with the PfCsx15 (0-5 μM dimer) and the cA_3 (2 and 5 μM, respectively) at 35°C for 15 min, before adding DNase VmeNucC (250 nM trimer). The fluorescence signal was plotted against time. **B.** Plot of baseline-corrected fluorescence signal against the ratio of PfCsx15 to cA_3. The average fluorescence signal from 49-51 min was corrected using the average baseline signal from 9-10 min. Data of replicates are presented as mean ± s.d. The DNase activity of NucC was inhibited only when PfCsx15 was present at the highest concentration.

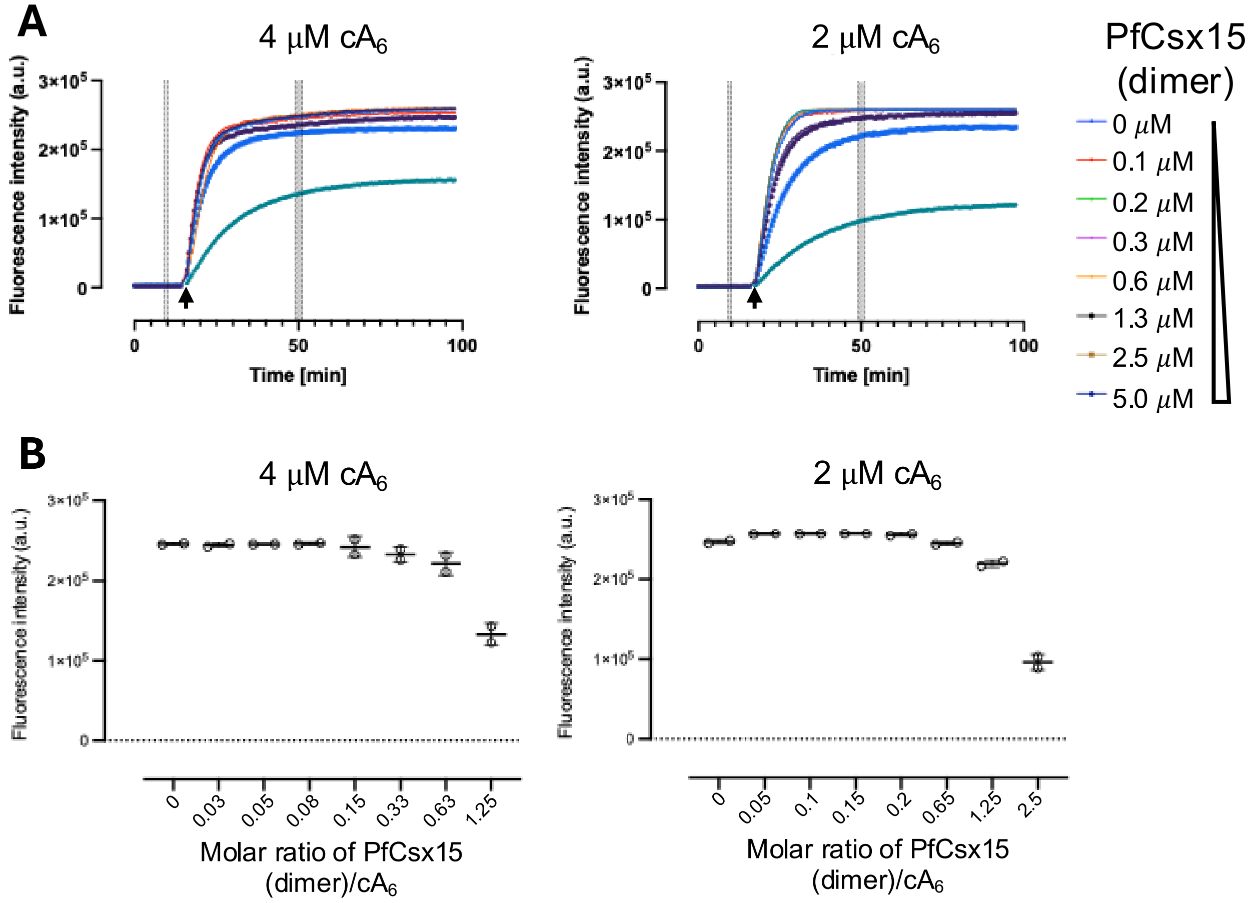

Supplementary Figure 2. PfCsx15 does not strongly inhibit the cA_6-induced effector MtbCsm6

A. Fluorescence signal emitted by RNA cleavage when RNases were presented. RNaseAlert substrates (100 nM) were incubated with the Csx15 (0-5 μM dimer) and the cA_6 (2 and 4 μM, respectively) at 35 °C for 15 min, before adding RNase MtbCsm6 (160 nM dimer). The fluorescence signal was plotted against time. **B.** Plot of baseline-corrected fluorescence signal against the ratio of PfCsx15 to cA_6. The average fluorescence signal from 49-51 min was corrected using the average baseline signal from 9-10 min. Data of replicates are presented as mean ± s.d. The RNase activity of Csm6 was partially inhibited only when PfCsx15 was present at the highest concentration.

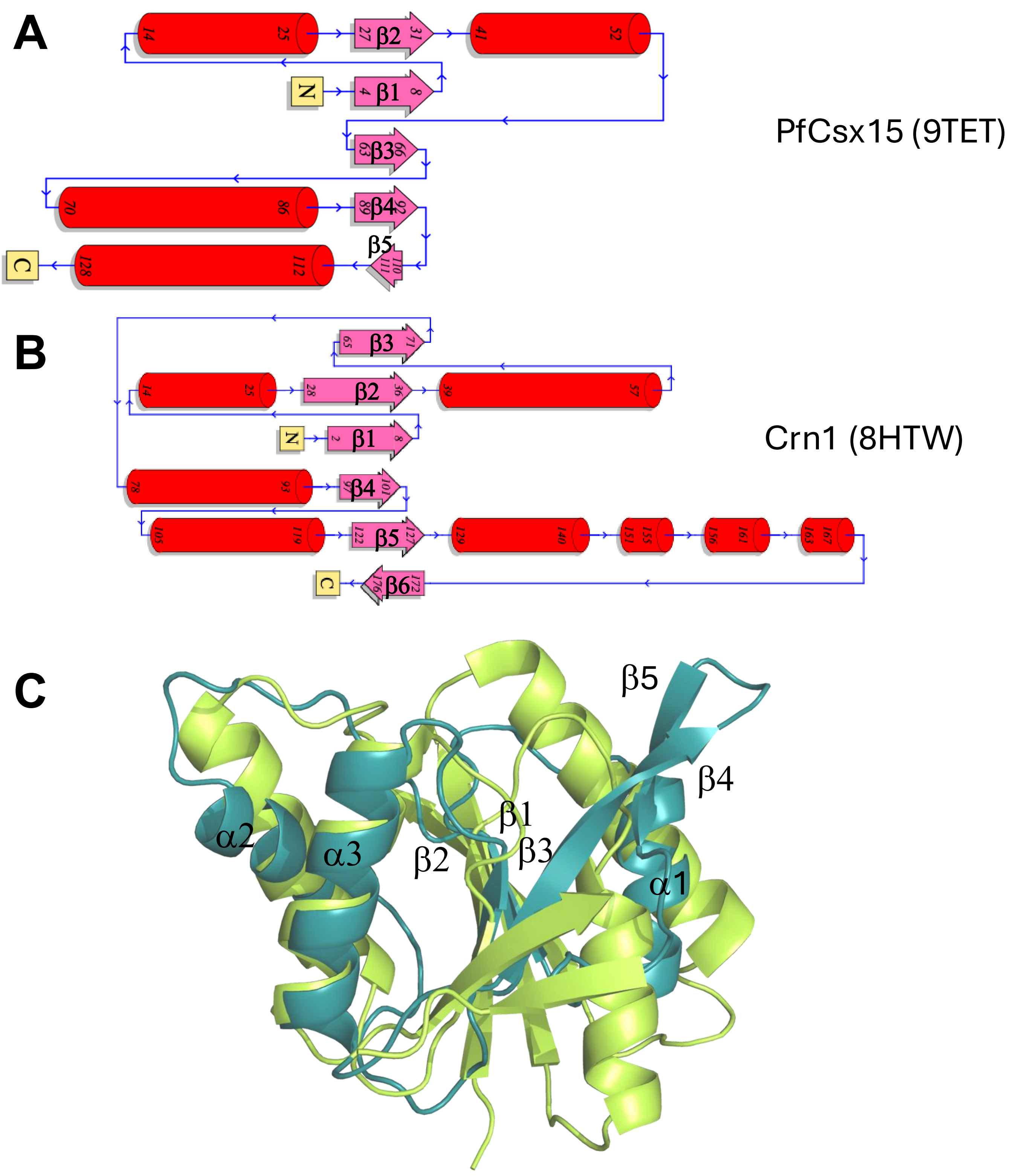

Supplementary Figure 3. Topological comparison of Csx15 and Crn1. Protein fold topology of PfCsx15 (**A**) and *Sulfolobus solfataricus* Crn1 (**B**), generated with PDBsum (https://www.ebi.ac.uk/thornton-srv/databases/pdbsum/), highlights the conserved Rossman fold architecture with a central 5/6 strand β-sheet flanked by α-helices. **C.** The core structures of PfCsx15 (teal) and Crn1 (yellow) align with an RSMD of 4.6 Å over 88 residues. Secondary structure elements in PfCsx15 are labelled.

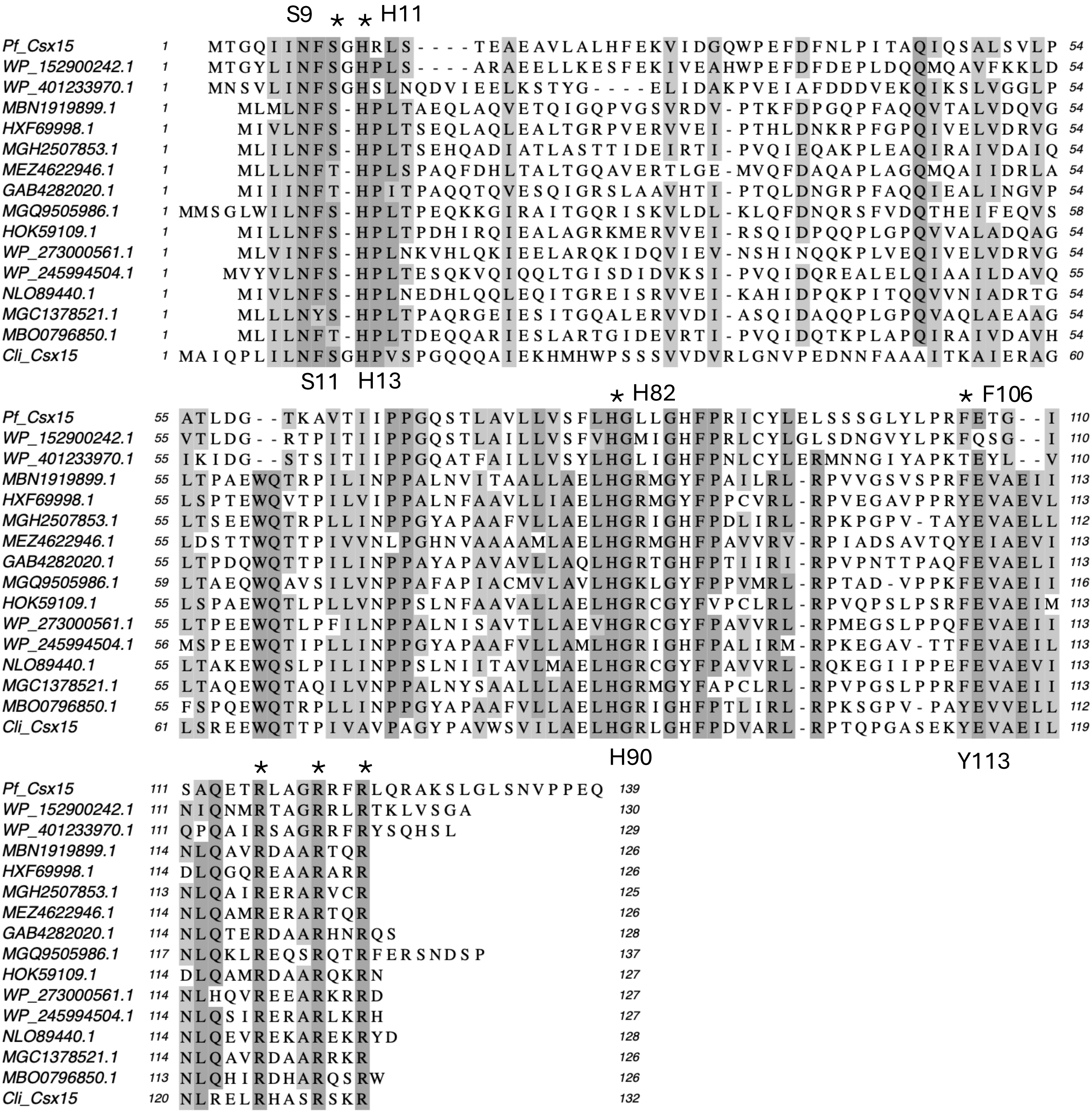

Supplementary Figure 4. Multiple sequence alignment of a range of Csx15 orthologues. Conservation is shown by shading and conserved residues mentioned in the text are indicated.

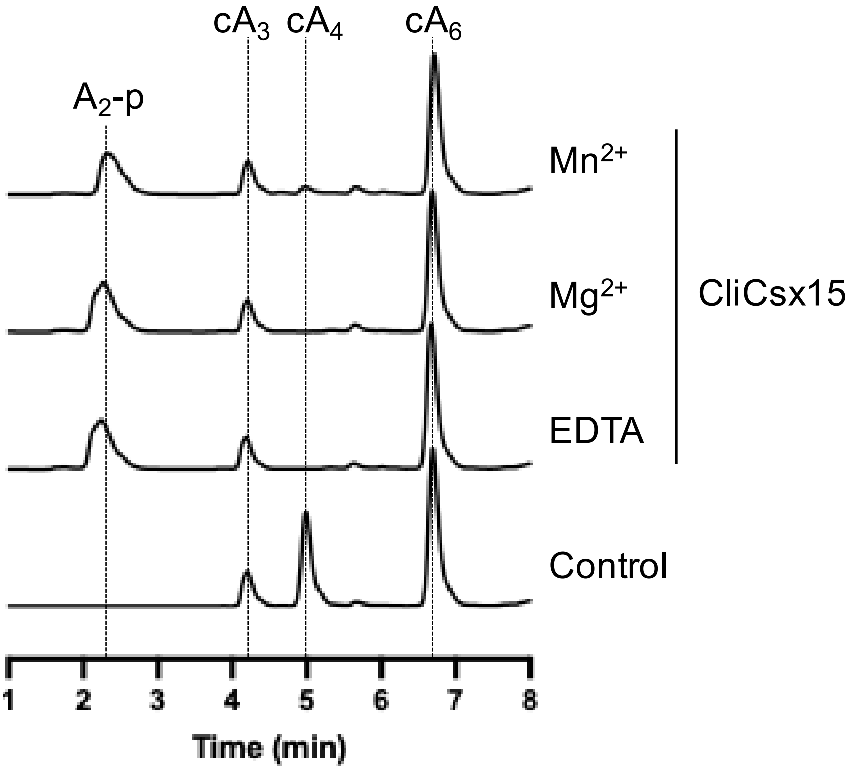

Supplementary Figure 5. Metal-independent ring nuclease activity of CliCsx15
HPLC analysis of CliCsx15 ring nuclease in the presence or absence of metals. The mixture of cA_3, cA_4 and cA_6 was incubated with equimolar CliCsx15 in the presence of 1 mM $MnCl_2$, $MgCl_2$ or EDTA, respectively, for 15 min at 30°C.

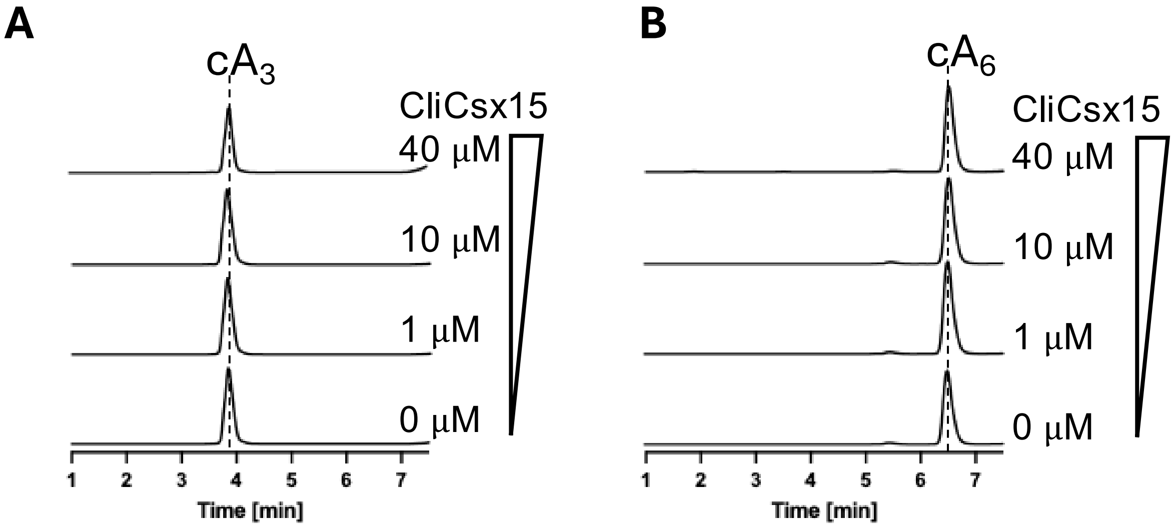

Supplementary Figure 6. CliCsx15 does not cleave cA_3 or cA_6

A. HPLC analysis of CliCsx15 with cA_3 and cA_6 in **B**. 80 μM cA_3 or cA_6 was incubated with CliCsx15 (0, 1, 10 and 40 μM) respectively, for 60 min at 30 °C.

A

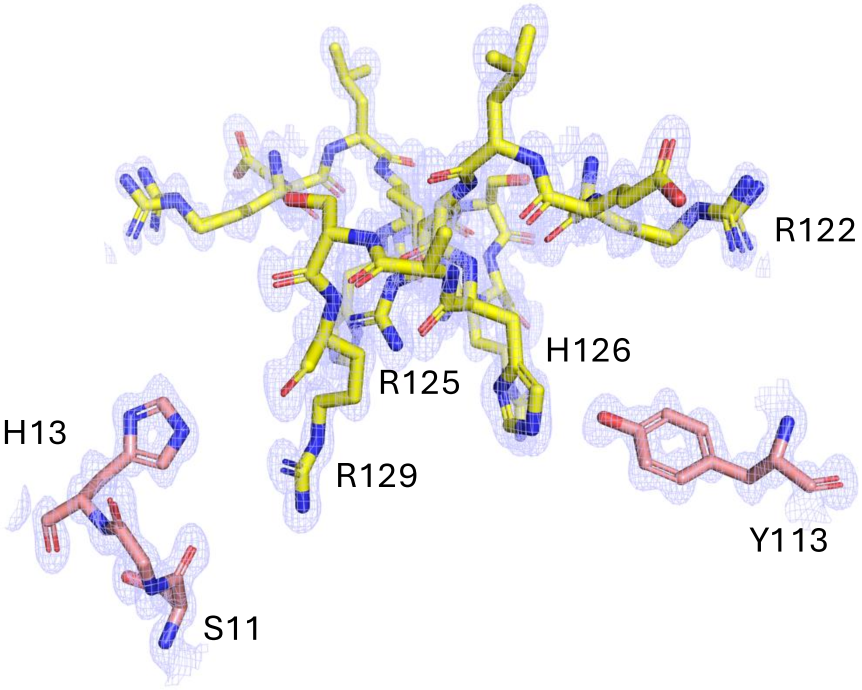

B

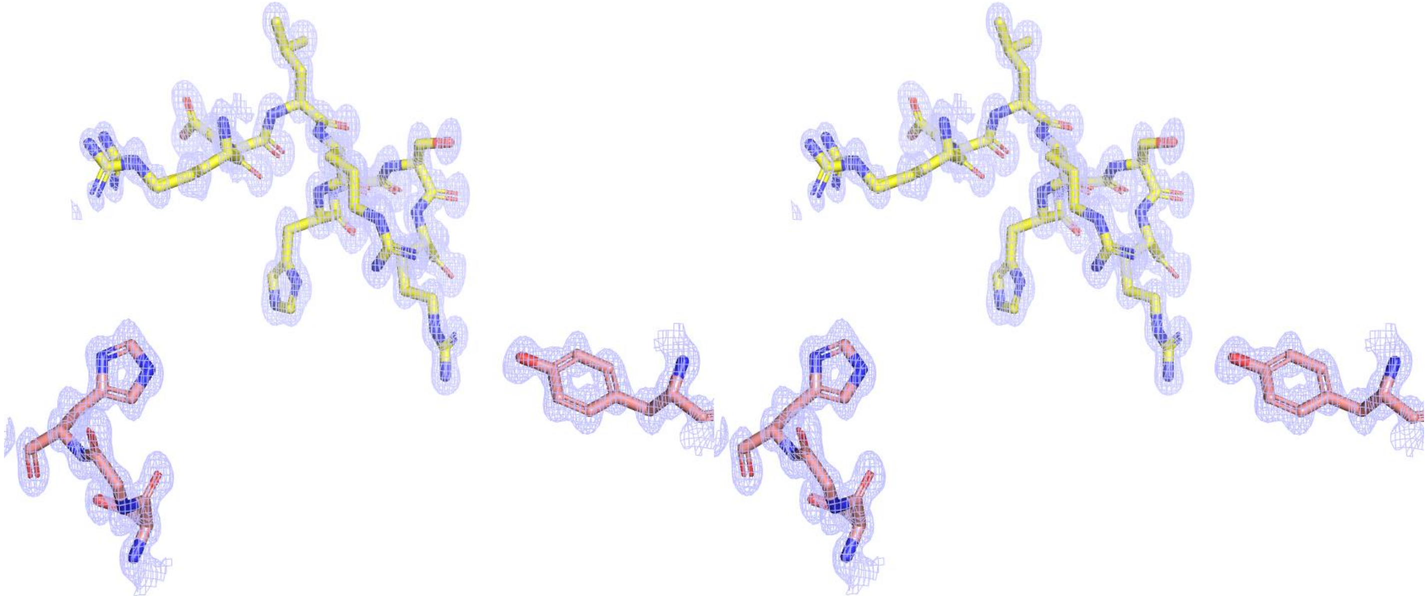

Supplementary Figure 7. 2Fo-Fc electron density map contoured at 1σ across the CliCsx15 dimer interface. A. Conserved residues are labelled. **B.** Shown as wall eyed stereo.

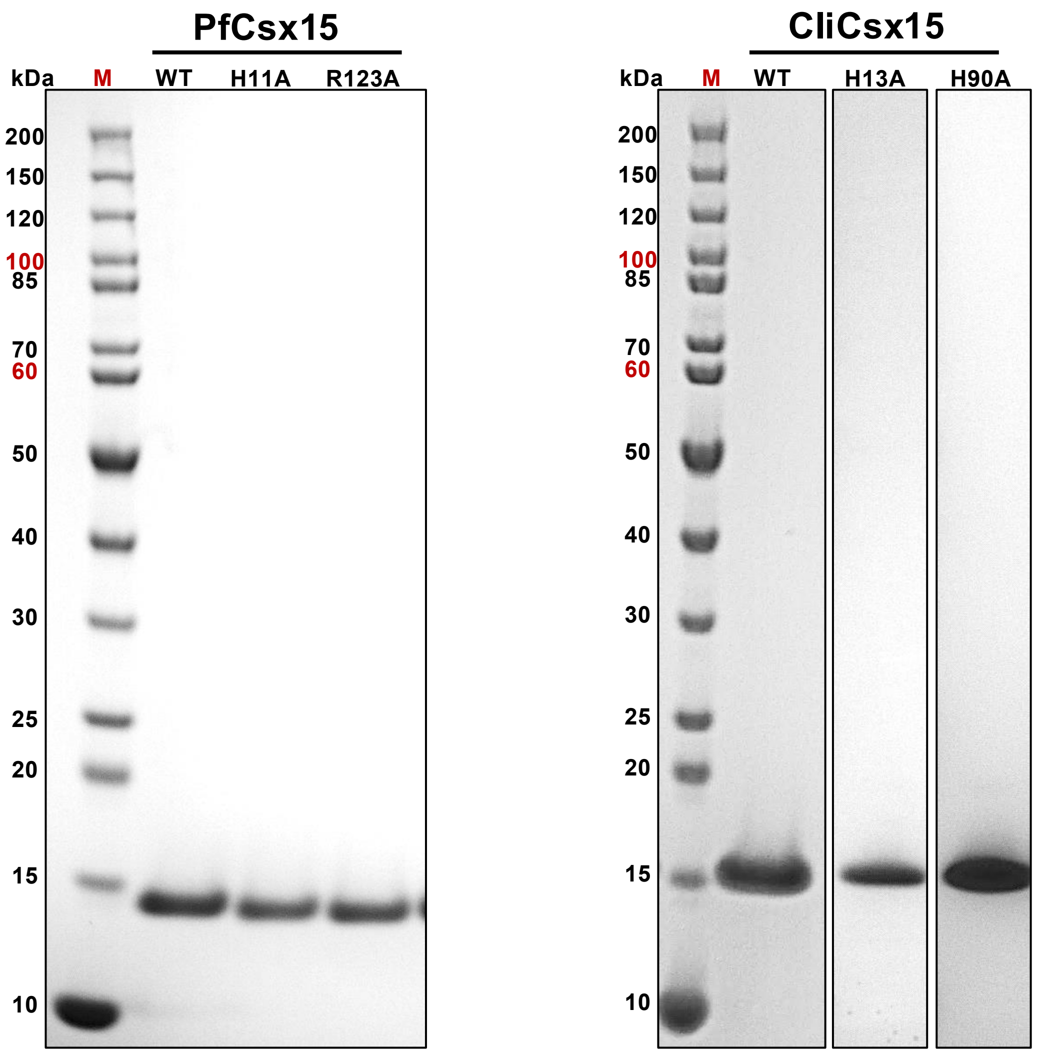

Supplementary Figure 8. SDS-PAGE analysis of Csx15 wild type and variants.

	CliCsx15	PfCsx15
**Data processing**		
Space group	C 1 2 1	P62
Cell dimensions		
a, b, c (Å)	83.3, 32.5, 57.8	89.3, 89.3, 31.4
α, β, γ (°)	90, 132.8, 90	90, 90, 120
Resolution (Å)	41.6 – 0.90 (0.92 – 0.9)	77.3 – 1.88 (1.91 – 1.88)
R_{merge}	0.036 (1.320)	0.135 (3.920)
I/σ(I)	16.8 (0.7)	18.2 (0.5)
Completeness (%)	94.6 (55.4)	99.8 (99.1)
Average redundancy<br>$CC_{1/2}$	6.2 (3.5)<br>1.000 (0.355)	40.0 (28.6)<br>1.000 (0.343)
V_m (Å^3/Da)	1.88	2.29
Solvent (%)	34.7	46.3
**Refinement**		
Unique reflections	79628 (2317)	11875 (582)
R_{work} / R_{free}	15.6 (34.0) / 16.8 (36.8)	19.8 (30.1)/ 24.6 (32.7)
Geometric deviations		
Bonds (Å) / Angles (°)	0.010 / 1.188	0.007 / 0.848
**No. atoms (non H)**		
Protein	1115	976
Water<br>PO_4<br>Glycerol	226	32<br>5<br>6
**B factors (Å^2)**		
Protein	11.2	53.2
Water<br>PO_4<br>Glycerol	23.2	53.9<br>51.7<br>61.8
Ramachandran<br>Favoured / outlier (%)	99.2 / 0	98.4 / 0
Molprobity score / centile (%)	0.86 / 98	1.04 / 100
Poor Rotamers	0	0
Favoured / outlier (%)	97.5 / 0	96.1 / 0
**PDB Code**	**9TEU**	**9TET**

* Values in parentheses are for the highest-resolution shell.

Supplementary Table 1. Crystallographic data collection and refinement statistics

Name	sequence (5'-3')	Notes
**Csx15** *Pseudomonas fluorescens* WP_191946534.1	GCGCCCATGGCACATATGACGGGTCAAATCATCAATTTTTCT<br>GGCCACCGTCTGTCCACTGAAGCGGAGGCCGTACTTGCGCT<br>TCATTTTGAGAAAGTCATTGACGGTCAGTGGCCCGAGTTTGA<br>CTTTAATCTGCCGATCACCGCTCAAATCCAATCAGCGTTGTC<br>AGTTTTGCCCGCAACATTAGACGGCACAAAGGCGGTTACCAT<br>CATTCCCCCTGGCCAGTCGACCCTTGCGGTCTTACTGGTGTC<br>CTTCTTACACGGTTTACTGGGGCATTTCCCGCGTATCTGCTA<br>CCTGGAGCTGTCGTCCTCCGGCCTTTATTTACCCCGCTTTGA<br>GACTGGTATTTCCGCCCAAGAGACGCGTTTGGCTGGCCGTC<br>GTTTCCGCTTACAACGCGCGAAGTCATTGGGCCTGTCGAATG<br>TACCTCCTGAGCAGTGACTCGAGGGATCCCGCG	g-Block
**Csx15** *Chlorobaculum limnaeum* WP_069809204.1	GCGCCCATGGCACATATGGCTATTCAACCGCTTATTTTAAACT<br>TTTCGGGACACCCAGTGTCACCCGGACAGCAACAAGCTATC<br>GAGAAACACATGCATTGGCCGTCCAGTAGCGTGGTGGACGT<br>TCGTCTGGGGAATGTCCCAGAGGATAATAACTTTGCTGCTGC<br>AATCACCAAAGCAATTGAACGCGCTGGGTTGTCACGCGAAGA<br>ATGGCAGACAACGCCAATCGTTGCCGTTCCGGCTGGCTACC<br>CAGCCGTCTGGTCGGTGATTCTGGCCGAGTTACATGGTCGC<br>CTTGGCCATTTTCCGGACGTAGCCCGCTTGCGCCCTACACA<br>GCCTGGGGCGTCCGAGAAGTACGAAGTTGCTGAAATTTTGAA<br>TCTGCGTGAGCTGCGCCACGCATCGCGCTCTAAGCGTTGAC<br>TCGAGGGATCCCGCG	
PfCsx15H11A-Fw	CAATTTTTCTGGCGCCCGTCTGTCCACTG	Mutagenesis Primers
PfCsx15H111A-Rv	CAGTGGACAGACGGGCGCCAGAAAAATTG	
PfCsx15R123A-Fw	GCCGTCGTTTCGCCTTACAACGCGCGAAG	
PfCsx15R123A-Rv	CTTCGCGCGTTGTAAGGCGAAACGACGGC	
CliCsx15H13A-Fw	CTTATTTTAAACTTTTCGGGAGCCCCAGTGTCAC	
CliCsx15H13A-Rv	GCTGTCCGGGTGACACTGGGGCTCCCGAAAAG	
CliCsx15H90A-Fw	GATTCTGGCCGAGTTAGCTGGTCGCCTTGGCC	
CliCsx15H90A-Rv	GGCCAAGGCGACCAGCTAACTCGGCCAGAATC	

Supplementary Table 2. Synthetic genes and mutagenesis primers for Csx15
